# Seven microbial bio‐processes to help the planet

**DOI:** 10.1111/1751-7915.12816

**Published:** 2017-08-03

**Authors:** Víctor de Lorenzo

**Affiliations:** ^1^ Centro Nacional de Biotecnología (CNB‐CSIC) Systems Biology Program 28049 Campus de Cantoblanco‐Madrid 28049 Spain

## Abstract

There is a number of global environmental problems that could be tackled through advanced Microbial Biotechnology.

At the onset of contemporary environmental awareness in the early 80s of last century, frequent triggers of public alarm included the spills of xenobiotic chemicals, the accidents of petroleum tankers and the bad quality of air and water that could be traced to unchecked industrial emissions. Episodes like the release of methyl isocyanate gas (and other chemicals in Bhopal (1984), the Exxon Valdez oil spill off the coast of Alaska (1989) or the heritage of TCDD)^1^ in soil left by the use of the agent orange^2^ in the Vietnam war were just a few of the cases that received widespread media exposure – and made society to demand solutions for combining economic growth with environmental quality. Together with many other incidents involving toxic pollutants, such events raised a considerable interest in understanding the fate of such man‐made (or human‐mobilized) molecules and the pursuit of options for managing them before, during and after their accidental or deliberate release. Note that the same decade witnessed the takeoff of recombinant DNA technology, which included the possibility of designing microorganisms for biodegradation (and eventually *in situ* bioremediation) of pollutants created by industrial and urban activities. The dividends of the science and research that environmental concerns of the time fostered have been immense. New chemicals are now subject to rigorous controls, many manufacturers have moved into eco‐friendly biocatalytic processes, risk assessment is now commonplace in the chemical industry, and the precautionary principle is applied to basically any new molecule with potential uses. Furthermore, there is a growing recycling culture along with much improved methods for disposing, treating and (bio)remediating chemical waste. Although incidents involving chemical spills will surely occur again, the experience makes us be much better prepared to deal with them should (and when) they happen. Fortunately, also, the wealth of knowledge on microbial metabolic pathways along with the possibility of developing biosensors and quickly assembling new catabolic routes for new compounds ease the management of what in the past were intractable environmental disasters. Multiscale modelling of geological and biological processes to predict the fate of chemical species of interest is also in place to guide decisions regarding pollution occurrences (Finley *et al*., 2009; Hadadi *et al*., 2016). Finally, Systems and Synthetic Biology is allowing a fresh revisiting of some not‐yet‐solved challenges about deliberate release of engineered microbiological agents for *in situ* remediation of chemical contaminants (de Lorenzo, 2008). In sum, I would argue that owing to all the research on the microbial‐pollutant interplay started 30 years ago, we have now plenty of technical choices to deal successfully with the environmental consequences of site pollution by toxic waste – and their deployment has to do more with political decisions than with obstinate scientific problems.

Alas, the same environmental research momentum initiated in the 1980s that has cracked many molecule‐specific and site‐specific contamination issues has exposed also several still more phenomenal environmental calamities. Some of them need to be urgently tackled, as they threaten the very continuity of the global ecosystem of planet Earth. The first and most concerning of all involves the industrial, urban and agronomic emissions of CO_2_ and other greenhouse gases. Their rising levels during the last few decades and their link to climate change need little explanation. Unlike toxic, abrupt and site‐specific pollution episodes associated with chemical spills, CO_2_ emissions (accompanied of those of methane, nitrous oxide, ozone, chlorofluorocarbons and hydrofluorocarbons) are basically inconspicuous and slow, but play a key role in global warming and its catastrophic consequences. Some models argue that further increases in average temperatures will place the planet on the way to non‐reversible deterioration to the point of making Earth non‐habitable (Mora *et al*., 2017). Recent data indicate that simply limiting CO_2_ emissions will not solve the problem^3^ and therefore that new technologies (including bio‐based ones; Antonovsky *et al*., 2016; Erb and Zarzycki, 2016) are badly needed for active and efficient carbon capture – and very soon. One of the worst consequences of global warming is the expansion of arid lands that are lost for agriculture or other activities beneficial to humans. It is noteworthy that a marginal change in humidity of a given ecosystem may entirely change its biological functioning when key species (whether animal, plants or microorganisms) are sensitive to dearth of water.

But greenhouse gases are not the only global environmental ordeal. Plastic pollution of oceans (in particular polycarbonate, polystyrene, PET,^4^ low‐/high‐density polyethylene, polypropylene and PVC^5^) has emerged also in the last few years as a major problem that endangers marine life by interfering with trophic and reproductive chains (Galloway and Lewis, 2016; Lebreton *et al*., 2017). It is worth of note that a large proportion of plastic discharges into the oceans are non‐accountable (Cózar *et al*., 2014; Sole *et al*., 2017), which suggests the existence of microorganisms able to massively metabolize them. Alas, the number of microbial strains that have been isolated and characterized is very limited, the biodegradation process is very slow, and strategies for large‐scale remediation (other than mechanical removal of the floating plastics^6^) remain to be developed. In reality, any effort for biological elimination of plastics originates a dilemma: their partial degradation originates microplastics, which may exacerbate their impact in marine ecosystems. In addition, their large‐scale catabolism would produce CO_2_, which could intensify the emissions of this greenhouse gas. Therefore, one could also consider the other way: entertaining strategies to compact plastic waste and make it sink in a form as non‐degradable as possible (i.e. fostering a kind of artificial inertization). Odd as it may look at first sight, one should consider that microbial processing of CO_2_ into sinking polymers (e.g. transparent exopolymers produced by microalgae) is one of the major mechanisms of microbial capture of carbon in the Ocean. In the absence of enough evidence or suitable models to favour one approach or the other (i.e. biodegradation vs. inertization), it would be reasonable to explore both ways as possible choices for sound decision‐making.

A separate type of global chemical contaminants (which therefore asks for global interventions) include endocrine disruptors and pharmaceuticals. The former comprise molecules that at small doses interfere with the body's hormonal system with detrimental effects on humans and wildlife. Typical compounds include alkylphenols, bisphenols, DDT, PCBs, polybrominated diphenyl ethers, phthalates and perfluorooctanoic acid. Note the very diverse origins and applications of these compounds, from insecticides to flame retardants, cosmetics and plasticizers. Some of them may be released during biodegradation or mere weathering of plastics, while others may just be extensively liberated and maintained intact due to their recalcitrance to microbial action (Ying *et al*., 2003; Liu *et al*., 2009). The second type of such silent pollutants involves bioactive molecules produced by the pharmaceutical industry, in particular antibiotics and hormones. Whether one origin or the other, the effects of these compounds become growingly noticeable through their effect on the reproductive abilities of affected populations and in the pollution of both freshwater and marine ecosystems. Note that the key property of biological systems is reproduction: any change that reduces it can lead to extinction. The essentially extensive, global nature of this type of pollution demands the development not only of new microbial pathways for their effective removal, but also the elaboration of strategies for their global dispersion – perhaps inspired in gene drives or some type of self‐propagating, massive horizontal gene transfer. Antibiotic resistance often spreads worldwide just a few years after their first utilization. Couldn't we learn from their dissemination mechanisms for the sake of spreading environmentally beneficial traits through the global microbiome?

Finally, modern agriculture has brought its own pollution problems – directly or indirectly. A large share of agricultural productivity depends on human‐formulated nitrogen and phosphorus fertilizers. The whole intensive food‐production system relies on the Haber‐Bosch reaction for conversion of N_2_ to ammonia and on the extraction of soluble phosphates from a few existing mines in the entire planet. The first process involves a costly chemical reaction (Ritter, 2008), while the second entails a one‐way, ultimately non‐sustainable flow of phosphorus from the mines to the fields and then washing and non‐reversible dilution into aquatic ecosystems. Release of agricultural N and P into rivers and coastal waters leads to eutrophication, red tides and food poisoning. These N‐related and P‐related problems could be met by developing new ways of biological fixation of nitrogen and recovery of diluted phosphorus from the oceans or sediments. Moreover, the success of modern, intensive agriculture comes along with generation of very large amounts of lignocellulosic waste that is difficult to degrade and often ends being burned or accumulated in landfills (Guerriero *et al*., 2016). Despite a long history of research on bacterial and fungal biodegradation – even valorization of such agricultural waste, the complexity of the molecules at stake, lignin in particular, makes the current processes still very inefficient. Surely, the lignin matrix contains an amazing molecular landscape built with useful chemical blocks that remain to be exploited. But we are not there yet.

The paragraphs above briefly address a list of environmental challenges that we must face to hand over the planet in good shape to the next generations. But what can Microbial Biotechnology do to meet such trials? Table 1 summarizes what I would nominate *the seven bio‐processes to help the World*. None of the tasks is straightforward, and all of them will surely demand a considerable research effort and an immense scientific and technical creativity. Synthetic Biology can help enormously in these endeavours. But let us be optimistic: If we have covered in 30 years the way to the wealth of knowledge and technologies on biodegradation that we enjoy now, there no reason that we cannot do the same quantum leap to solve problems that look intractable at the time they are identified.

**Table 1:** Seven key microbial‐based processes to address global environmental problems

Num	Challenge	Possible Microbial Biotechnology avenues
1	Reverting atmospheric levels of CO_2_ and greenhouse gases	Development and global spreading^a^ of non‐photosynthetic CO_2_ capture pathways
2	Increasing humidity of arid ecosystems	Expression and spreading^a^ of H_2_O‐capturing proteins in desiccation‐resistant soil bacteria
3	Cleanup of plastic waste in marine ecosystems	Designing and spreading^a^ of bacterial pathways for complete mineralization of different plastic types
		Scheming and spreading^a^ of plastic‐flocculation/agglomeration surface adhesins^b^
4	Eliminating pharmaceuticals and endocrine disruptors from trophic chains	Assembly and spreading^a^ of specific pathways for complete biodegradation of each of the molecules at stake
5	Increasing biological fixation of nitrogen	Invention/evolution of bacterial O_2_‐insensitive routes of nitrogen fixation
		Expression of microbial N_2_‐fixation enzymes in plants
6	Recovery of diluted phosphorus from marine ecosystems and sediments	Engineering and spreading of phosphate hyperaccumulators
7	Management of lignocellulosic compounds	Construction of microbial strains tailored for complete de‐polymerization of lignin into building blocks
		Design and microbial expression of pathways for biosynthesis of recalcitrant lignin forms^b^

a The issue involves not only genetic assembly of all biological activities necessary to manifest the desired activity, but also its propagation through the environmental microbiome through either horizontal gene transfer or some type of prokaryotic gene drive, e.g. with engineered phages.

b In this case, the idea is not to degrade the compound, but just the opposite: making it recalcitrant to biodegradation and thus capture the cognate carbon in an inert form.

## Conflict of interest

None declared.

## Notes

1 2,3,7,8‐tetrachlorodibenzo‐p‐dioxin.

2 A formulation composed of two phenoxyl herbicides – 2,4‐dichlorophenoxyacetic acid (2,4‐D) and 2,4,5‐trichlorophenoxyacetic acid (2,4,5‐T).

3 https://goo.gl/NPRBkC.

4 Polyethylene terephthalate.

5 Polyvinyl chloride.

6 https://www.theoceancleanup.com/.
